# Integrated chemometric fingerprints of antioxidant activities and HPLC–DAD–CL for assessing the quality of the processed roots of *Polygonum multiflorum* Thunb. (*Heshouwu*)

**DOI:** 10.1186/s13020-016-0087-8

**Published:** 2016-04-12

**Authors:** Hai Fang Chen, You Hua Chen, Chun Hua Liu, Lu Wang, Xi Chen, Bo Yang Yu, Jin Qi

**Affiliations:** Jiangsu Key Laboratory of TCM Evaluation and Translational Research, China Pharmaceutical University, 639 Longmian Road, Nanjing, 210009 China; Key Laboratory of Modern Preparation of TCM of Ministry of Education, Jiangxi University of Traditional Chinese Medicine, Nanchang, 330004 China; Department of Complex Prescription of Traditional Chinese Medicine, State Key Laboratory of Natural Medicines, China Pharmaceutical University, 639 Longmian Road, Nanjing, 211198 China

## Abstract

**Background:**

The processed roots of *Polygonum multiflorum* Thunb. (*Heshouwu*; processed HSW) are commonly used in anti-aging medicine. Few reports have combined chemical profiles with bioactivity to evaluate the quality of the processed HSW. This study aims to integrate chemometric fingerprints of antioxidant activities and high-performance liquid chromatography–diode array detection–chemiluminescence (HPLC–DAD–CL) to assess the quality of processed HSW.

**Methods:**

An online HPLC–DAD–CL based on the three reactive oxygen species (ROS), superoxide anion, hydrogen peroxide, and peroxynitriteanion, was developed to screen the potential anti-aging constituents for a comprehensive quality evaluation of processed HSW. Additionally, antioxidant-activity-integrated fingerprints were constructed and hierarchical cluster analysis and principal component analysis were used to evaluate the variations among 14 batches of processed HSW samples purchased from drug stores in different habitats.

**Results:**

Fourteen batches of processed HSW samples were highly similar and classified into two clusters using hierarchical cluster analysis. Twelve active compounds exhibited antioxidant activity on the ROS with different degrees of sensitivity that constituted specific fingerprints. Among them, protocatechuic acid, catechin, *trans*-2,3,5,4′-tetrahydroxy-stilbene-2-O-β-d-glucoside, 2,3,5, 4′-tetrahydroxy-stilbene-2-O-β-d-(2′′-galloyl)-glucoside, torachrysone-8-O-glucoside, and emodin-8-O-β-d-glucoside exerted relatively large influences on the differences between processed HSW samples.

**Conclusion:**

Our study established the antioxidative activity-integrated fingerprint for processed HSW and achieved a screening of the potential anti-aging constituents using the online HPLC–DAD–CL method with H_2_O_2_, O_2_^•−^, and ONOO^−^scavenging experiments.

**Electronic supplementary material:**

The online version of this article (doi:10.1186/s13020-016-0087-8) contains supplementary material, which is available to authorized users.

## Background

**Fig. 1:**
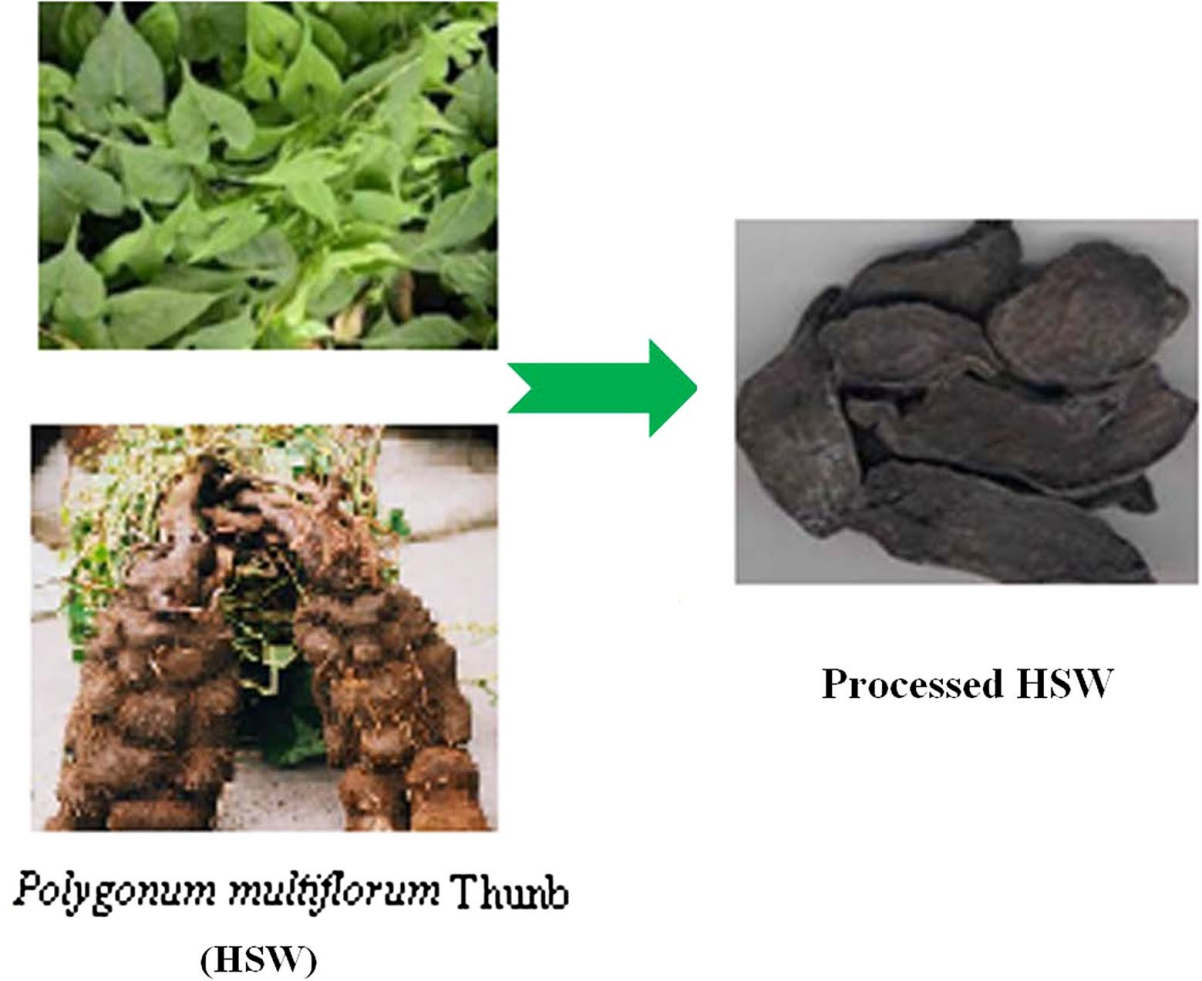
Photos of *Polygonum multiflorum* Thunb.

The roots of *Polygonum multiflorum* Thunb. (*Heshouwu*; HSW) (Fig. 1) are often used in either raw or processed form to treat different diseases in Chinese medicine (CM). Raw HSW loosens the bowel and relieves constipation; HSW’s anti-aging property is mainly attributed to the processed form [1]. HSW also exhibits neuroprotective [2] and hairgrowth promoting activity [3, 4]. The characteristic constituent of HSW is 2,3,5,4′-tetrahydroxystilbene-2-O-β-d-glucoside, which is a potential natural inhibitor of advanced glycation end products [5] and exhibited anti-atherosclerosis [6] and anti-osteoporosis effects [7].

HSW contains numerous phenols, anthraquinones, and stilbene glycosides [8, 9]. Most studies have used various methods to focus on qualitative or quantitative analysis of HSW constituents, including high-performance liquid chromatography (HPLC) [10, 11], HPLC–electrospray ionization mass spectrometry (HPLC–ESI–MS) [12], and UHPLC (ultra-HPLC) with linear iontrap–Orbitrap tandem mass spectrometry (UHPLC–LTQ–Orbitrap MS) [13]. However, none of these studies have comprehensively evaluated the quality of HSW by combining chemical components with bioactivity. In addition, research has focused on studies of raw HSW rather than processed HSW. Different processing procedures used by manufacturers could lead to differences in the quality of processed HSW [14]. Therefore, it is necessary to analyze chemical and bioactivity information to comprehensively control the quality of processed HSW.

The fingerprint technique is the predominant tool to evaluate the quality of CM. However, the chromatographic fingerprint only contains the chemical message and is insufficient to demonstrate the overall quality of material [15, 16]. Therefore, activity-integrated fingerprints have been increasingly used to evaluate the quality of CM. High-performance liquid chromatography–diode array detection–chemiluminescence (HPLC–DAD–CL) is a sensitive method and often used in studies of activity-integrated fingerprints. Some studies have used a series of free radicals, such as hydrogen peroxide (H_2_O_2_), 2,2′-Azinobis-(3-ethylbenzothiazoline-6-sulfonic acid), superoxide anion (O_2_^•−^), and 2, 2-diphenyl-1-picrylhydrazyl, to screen bioactive constituents and evaluate the antioxidant activity of CM [15–17]. However, the bioactive constituents obtained using these methods by means of their scavenging activity on one free radical could be insufficient because of their different scavenging capacity on different free radicals. Therefore, it is necessary to develop a multi-free radical scavenging system to obtain chemical and bioactive information to evaluate the quality of CM.

Aging is a complex physiological process and the oxidative stress theory of aging has gained considerable support [18].Numerous studies indicate that reactive oxygen species (ROS), such as O_2_^•−^, H_2_O_2_, and peroxynitriteanion (ONOO^−^), are involved in the aging process and cause oxidative damage [19–22]. Antioxidative activity may be one index of the anti-aging effect. The anti-aging effect of HSW has been studied in pharmacological experiments [4, 23], but the search for anti-aging constituents is time-consuming, particularly because the content of constituents from different habitats varies markedly. Therefore, the selection of characteristic chemical markers using the HPLC–DAD–CL method may be a faster way of comprehensively evaluating the quality of HSW.

This study aims to investigate the antioxidant profile of processed HSW by HPLC–DAD–CL combined with chemometrics to rapidly screen potential anti-aging constituents of processed HSW by scavenging three reactive species (O_2_^•−^, H_2_O_2_, and ONOO^−^).

## Methods

### Materials and reagents

HPLC grade acetonitrile was obtained from Tedia (Tedia Company Inc., USA). Luminol (Fluka Chemie Buchs, Switzerland), hydrogen peroxide solution (30 % H_2_O_2_ in water), sodium nitrite (NaNO_2_), sodium carbonate (Na_2_CO_3_), sodium bicarbonate (NaHCO_3_), and hydrochloric acid (HCl) were all purchased from Nanjing Chemical Regent Corporation (Jiangsu, China). Pyrogallol was obtained from Zunyi Second Chemical Corporation (Guizhou, China). Ethylenediaminetetraacetic acid was supplied by Shanghai Chemical Reagent Corporation (Shanghai, China). Sodium hydroxide (NaOH) and manganese dioxide (MnO_2_) were purchased from Xilong Chemical Corporation (Guangzhou, China). The reagents used were all of analytical grade. The purified water used was prepared by a Millipore water purification system (Millipore, Bedford, MA, USA).

### Preparation of samples

Fourteen batches of processed HSW samples were purchased from different drug stores. The habitats of samples were as follows: Guangdong (20110901, S1), Shanxi (20110702, S2), Hebei (20080526, S3), Guizhou (20080323, S4), Yunnan (20090327, S5), Anhui (20061228, S6), Guangdong (20090705, S7), Hubei (20110523, S8), Sichuan (20110419, S9), Sichuan (20091216, S10), Henan (20120615, S11), Guangxi (20120530, S12), Guizhou (20121129, S13), and Hunan (20120803, S14). All samples were authenticated by Professor Bo-Yang Yu based on their morphological features according to the Chinese Pharmacopoeia [24]. Their voucher specimens were deposited at the Department of Complex Prescription of CM, China Pharmaceutical University, Nanjing, China. Processed HSW samples were ground in a grinder producing a 60-mesh particle size powder. Each sample (1.0 g) was accurately weighed and extracted twice with 50 mL methanol for 30 min in an ultrasonic bath. Then, the extract was vacuum filtered each time. Extraction solutions were mixed together after cooling and evaporated under vacuum at 40 °C. The residue was diluted with methanol (10 mL).The sample solution was further filtered through a 0.22-µm membrane filter prior to injection into the HPLC–CL system.

### Preparation of CL solutions

Carbonate buffers (pH 10.0 and 11.0; 0.1 M) were prepared by mixing appropriate volumes of 0.1 M Na_2_CO_3_ and 0.1 M NaHCO_3_ solution. A 1.8 × 10^−2^ M stock solution of luminol was prepared in a 0.1 M Na_2_CO_3_ solution and stored in a refrigerator for at least 3 days before dilution. A 1.0 × 10^−2^ M stock solution of pyrogallol was prepared in a 0.1 mM HCl solution and then stored in a dark bottle at 4 °C. Reagent solutions for the determination of H_2_O_2_, and O_2_^•−^ scavenging activity were prepared according to previous work conducted by our research group [15]. Peroxynitrite was prepared according to a previous method [25] and we used an online peroxynitrite scavenging detection method developed by our group [26]. Reagent solutions for the determination of peroxynitrite scavenging activity were as follows: a 9 × 10^−6^ M luminol solution (Solution I) was prepared with 0.1 M carbonate buffer solution (pH 9, NaHCO_3_: Na_2_CO_3_ = 9:1) and a 4.97 × 10^−4^ M peroxynitrite solution (Solution II) was prepared with a 0.1 M NaOH solution on an ice bath.

### HPLC–DAD–CL analysis condition

The HPLC system used was a Shimadzu LC-2010C HT system consisting of a quaternary pump, an autosampler (Shimadzu Corporation, Japan), a thermostated column compartment (Shimadzu Corporation, Japan), and a DAD (SPD-M20A, Shimadzu Corporation, Japan). HPLC separation was achieved with a Venusil MP C_18_ column (250 × 4.6 mm, 5 μm, Agela Technologies, China) with a flow rate of 1.0 mL/min. The column temperature was set at 30 °C. The mobile phase was composed of A (acetonitrile) and B (0.1 % aqueous phosphoric acid, v/v). The gradient elution program was as follows: 0–6 min, isocratic gradient 6 %A; 6–10 min, linear gradient 6 %–10 %A; 10–30 min, linear gradient 10 %–20 %A; 30–70 min, linear gradient 20 %–50 %A; 70–75 min, linear gradient 50 %–60 %A; 75–85 min, isocratic gradient 60 %A.

**Fig. 2:**
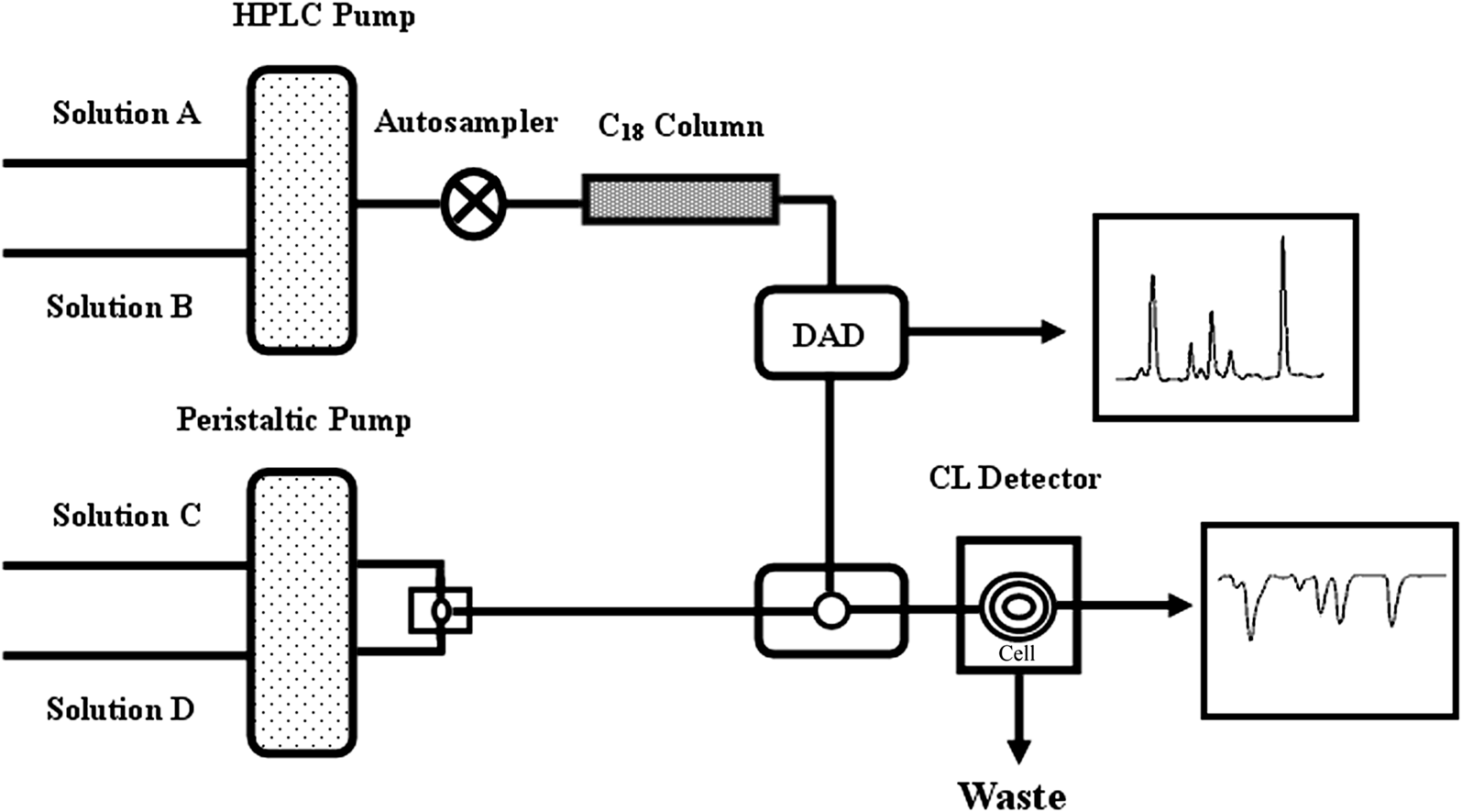
Detection apparatus for HPLC–DAD–CL

A BPCL ultraweak bioluminescence system (Academia Sinica Biophysics Institute, Beijing, China) was used to monitor the CL emission. The CL detector was equipped with a flat glass coil (80 µL) as detection cell and a photomultiplier operated at −800 V. The HPLC–DAD–CL detection system was interconnected with PEEK tubes (Mianyang Prochema Commercial Corporation, China). CL reagents, luminal solutions, H_2_O_2_, pyrogallol, and peroxynitrite were transported by peristaltic pumps at the same flow rate of 1.1 mL/min each. CL reagents were mixed with sample solution eluted from the HPLC detector then passed through the CL detector; the CL emission was detected by the BPCL system (Fig. 2). Resveratrol is a strong, natural antioxidant, therefore, the overall antioxidant activities for processed HSW were evaluated using resveratrol as a positive control (a similar method has been reported in the literature [15]). The activity of the antioxidant constituents in processed HSW was proportional to the intensity of negative peak and evaluated with the scavenging rate (%), as shown in the following Eq. (1):

1 $$ {\text{Scavenging rate }}\left( {\text{\% }} \right) = \frac{{{\text{CL}}_{0} - {\text{CL}}_{1} }}{{{\text{CL}}_{0} }} \times 100, $$

where CL_0_ is the baseline intensity of CL (without sample) and CL_1_ is the inhibited CL intensity of every compound in the extracts.

### HPLC–ESI–MS analysis condition

An Agilent 1100LC/MSD Trap XCT ESI (Agilent Technologies, MA, USA) was used to obtain information about the structure of the constituents in processed HSW. The mobile phase for HPLC–MS was the same as described for the HPLC analysis conditions but the composition of the B pump substituted 0.1 % aqueous phosphoric acid for 0.1 % aqueous formic acid. The ESI–MS spectra were acquired in negative ionization mode. Capillary voltage was 3300 V. Drying gas temperature was set at 350 °C with a flow rate of 9.0 L/min and nebulizing pressure was set at 275.8 kPa. Data were processed using the 6300 Series HPLC/MSD Trap and Data Analysis 3.4 (Agilent Technologies, MA, USA).

### Data analysis of chromatographic profiles and ROS scavenging activity of processed HSW samples

The characteristic constituent in processed HSW samples was selected as the reference peak to calculate relative retention time (RRT) and relative peak area (RPA). The relative standard deviations (RSDs) of RRT and RPA for each common peak were calculated to estimate precision, repeatability, and stability. The method precision was evaluated using intraday and interday variation tests. The intraday variation test was evaluated using five replicate injections of the same sample and the interday variation test was evaluated over 3 days with five replicate injections each day. Method repeatability was analyzed for the six replicate samples. The stability of the sample solution was evaluated at different time points in one day (0, 2, 4, 8, 12, and 24 h).

We used a similarity evaluation system for chromatographic fingerprints of CM (Version 2004 A) to evaluate the similarity of the samples. Six samples from different places were prepared. The similarities between the chromatograms of the samples from different origins were examined by comparing the samples with the reference fingerprint that was generated by the median value of all the chromatograms.

### Statistical analysis

Hierarchical cluster analysis (HCA) was performed by SPSS statistics software (SPSS for Windows 11.5, SPSS Inc., Chicago, USA) was used to sort the samples into different groups. The distance between the samples was used to assess the similarities among processed HSW samples. Samples with high similarity were clustered into homogenous groups. Principal components analysis (PCA) is an effective method to determine the main factors in large amounts of data by feature extraction and dimensionality reduction. PCA analysis of the fingerprint data of processed HSW was performed using SIMCA-P 12.0 software (Umetrics AB, Umea, Sweden).

Data were expressed as the mean ± standard deviation (SD). The statistical significance of differences between means was established by One-way ANOVA with Turkey post hoc tests. Significant differences and highly significant differences were classed as values of *P* < 0.05 and values of *P* < 0.01, *P* < 0.001, respectively.

## Results and discussion

### HPLC fingerprint method

*Trans*-2,3,5,4′-tetrahydroxy-stilbene-2-O-β-d-glucoside (peak 9) was selected as the reference peak. The RSDs of RRT and RPA for the precision of each common peak ranged from 0.51 to 2.33 % (n = 6) in the intraday variation test, respectively, and ranged from 0.83 to 3.97 % (n = 5) in the interday variation test, respectively. The RSDs for the repeatability of RRT and RPA of each common peak ranged from 0.9 to 4.1 % (n = 5), respectively. The RSDs of the stability test were below 2.8 % (n = 6) (Additional file1).

### Identification of the main constituents in processed HSW by HPLC–ESI–MS

The structures of compounds in processed HSW were identified by comparing retention time and MS data with previous findings [13, 27–30]. The structures of these constituents were further confirmed using available reference standards, including gallic acid, protocatechuic acid, catechin, epicatechin, *trans*-2,3,5,4′-tetrahydroxy-stilbene-2-O-β-d-glucoside, emodin, physcion-8-O-β-d-glucoside, and emodin-8-O-β-D-glucoside (Additional file 2). The MS data are shown in Table 1.

**Table 1:** Characterization of chemical constituents of the processed HSW samples

Peaks	*T*R (min)	Formula	MS (m/z)	MS^n^ (m/z)	Identification	λ_max_ (nm)
1	8.20	C_7_H_6_O_5_	168.8[M-H]^−^	124.8 [M-H-CO_2_]^−^,	Gallic acid	270
2	13.262	Unknown	323.1 [M-H]^−^	–	Unknown	285
3	14.134	C_7_H_6_O_4_	152.8 [M-H]^−^	108.8 [M-H-CO_2_]^−^	Protocatechuic acid	260, 293
4	18.582	Unknown	185.8 [M-H]^−^	141.8 [M-H-CO_2_]^−^	Unknown	218, 279
5	21.664	C_15_H_14_O_6_	289.0 [M-H]^−^	244.8 [M-H-CO_2_]^−^	Catechin	278
6	26.645	C_15_H_14_O_6_	289.0 [M-H]^−^	244.9 [M-H-CO_2_]^−^	Epicatechin	219, 282
7	29.50	C_26_H_32_O_14_	567.1 [M-H]^−^	613.2 [M-H + HCOO]^−^, 405, 242.9	2, 3, 5, 4′-tetrahydroxystilbene-2-O-(6′′-O-α-d-glucopyranosyl)-β-*D*-glucopyranosyl)	320
8	30.28	C_20_H_22_O_9_	405.1 [M-H]^−^,	441 [M-H + Cl]^−^, 242.8[M-H-glu] ^−^	*Cis*-2, 3, 5, 4′-tetrahydroxy-stilbene-2-O-β-d-glucoside	283
9	37.17	C_20_H_22_O_9_	405.1 [M-H]^−^,	441 [M-H + Cl]^−^, 242.8[M-H-glu] ^−^	*Trans*-2, 3, 5, 4′-tetrahydroxy-stilbene-2-O-β-d-glucoside	213, 322
10	38.112	C_22_H_18_O_10_	441.1 [M-H]^−^	288.9, 330.9	Unknown	273
11	38.538	C_27_H_26_O_13_	557.2 [M-H]^−^	312.9	2, 3, 5, 4′-tetrahydroxy-stilbene-2-O-β-d-(2″- galloyl)-glucoside	214, 293
12	49.58	C_20_H_24_O_9_	407.1 [M-H]^−^	244.9 [M-H-glu]^−^	torachrysone-8-O-glucoside	324
13	50.74	C_21_H_20_O_10_	431.1 [M-H]^−^	268.9 [M-H-glu]^−^	Emodin-8-O-β-d-glucoside	222, 282,422
14	55.64	C_22_H_22_O_10_	283 [M-H-glu]^−^	444.9 [M-H]^−^, 239.9 [M-CH_3_-CO]^−^, 240.9	Physcion-8-O-β-d-glucoside	222, 271,422
15	69.80	Unknown	283	239.8	Unknown	224, 285,434
16	79.79	C_15_H_10_O_5_	268.9 [M-H]^−^	224.8 [M-H-CO_2_]^−^	Emodin	287, 439
17	9.0	Unknown	–	–	–	
18	33.09	Unknown	–	–	–	

*–* Not detected by HPLC–ESI–MS

### Antioxidant activity of processed HSW samples

The standard potency equation of Ln(*y*) and the scavengingrate (%) (*x*) for resveratrol scavenging O_2_^•−^, H_2_O_2_, and ONOO^−^ in different concentrations were obtained. The equations for resveratrol scavenging the H_2_O_2_, O_2_^•−^, and ONOO^−^ were, respectively, as follows: Ln(*y*) = 1E − 05x^3^ − 0.0015x^2^ + 0.1083x − 6.775(R^2^ = 0.9997, *P* < 0.001), Ln(*y*) = 2E − 05x^3^ − 0.0032x^2^ + 0.2001x − 6.0478 (R^2^ = 0.9992, *P* < 0.05), Ln(*y*) = 1E − 05x^3^ − 0.0022x^2^ + 0.1373x − 6.3504(R^2^ = 0.9973, *P* < 0.001). If resveratrol (1 µg) was a potency unit, the relative total activities should be the sums of peak potency. The relative total activities of peaks in 14 batches of processed HSW were calculated according to Eq. (1). The results are shown in Tables 2, 3 and 4. Compared with S1 and S4 samples,the antioxidative activities of S10 and S11 in scavengin ROS were relatively low (*P* < 0.001) and the antioxidative activities of samples S2 and S12 exhibited insignificant differences (*P* = 1). The S2 and S12 samples had strong antioxidative activities, particularly in scavenging O_2_^•−^ and H_2_O_2_. The qualities and antioxidative effects of S7 in scavenging O_2_^•−^, H_2_O_2_ and ONOO^−^ were significantly different from S1, although the two samples were from Guangdong province (in scavenging O_2_^•−^: *P* = 0.037; in scavenging H_2_O_2_ and scavenging ONOO^−^: *P* < 0.001). The same was true for samples S4 and S13. Compared with S4, the antioxidative effects of S13 were obviously weak in scavenging O_2_^•−^, H_2_O_2_ and ONOO^−^ (*P* < 0.001). The detailed comparison data was shown in Additional file 3. Different processing technology may have caused the variation of chemical compositions and differences in bioactivity. This indicates the importance of the standardization of processing technology.

**Table 2:** The H_2_O_2_ scavenging rate of the constituents from fourteen batches of the processed HSW samples (n = 6)

No.	Peak 1	Peak17	Peak 3	Peak 5	Peak 8	Peak 18	Peak 9	Peak 11	Peak 12	Total activity (µg)
S1	18.25 ± 0.15	19.19 ± 0.93	32.28 ± 1.76	9.501 ± 0.32	8.89 ± 0.83	22.20 ± 0.17	96.83 ± 3.45	7.12 ± 0.16	16.16 ± 0.23	0.32
S2	22.58 ± 0.23	21.73 ± 1.05	13.30 ± 0.32	59.71 ± 0.67	25.71 ± 0.99	43.73 ± 0.35	94.21 ± 2.36	30.36 ± 0.91	59.29 ± 1.89	0.33
S3	19.78 ± 0.11	9.95 ± 0.51	18.47 ± 0.65	4.88 ± 0.77	17.65 ± 0.71	41.33 ± 0.27	93.09 ± 2.30	9.64 ± 0.66	46.23 ± 2.33	0.25^##^
S4	24.43 ± 0.09	28.72 ± 0.35	29.82 ± 0.89	64.59 ± 2.88	20.44 ± 0.63	31.33 ± 0.38	96.54 ± 4.50	33.40 ± 1.78	7.64 ± 0.59	0.36
S5	17.06 ± 0.05	18.01 ± 0.73	17.77 ± 1.01	11.63 ± 1.20	29.58 ± 0.58	31.17 ± 0.64	94.43 ± 1.19	7.02 ± 0.32	3.15 ± 0.09	0.26^#^
S6	6.87 ± 0.50	18.93 ± 0.45	12.80 ± 0.25	33.03 ± 1.76	33.27 ± 0.12	33.16 ± 0.47	93.34 ± 0.57	10.20 ± 0.38	25.21 ± 1.75	0.26^###^
S7	13.19 ± 0.17	12.04 ± 0.80	11.62 ± 0.17	32.90 ± 1.28	20.72 ± 0.11	21.10 ± 0.32	88.53 ± 1.35	5.69 ± 0.15	1.68 ± 0.18	0.17***^, ###^
S8	13.60 ± 0.34	22.21 ± 0.66	28.42 ± 0.03	10.72 ± 0.53	30.20 ± 0.27	21.84 ± 0.26	91.29 ± 4.11	4.14 ± 0.32	1.45 ± 0.12	0.21*^, ###^
S9	18.11 ± 0.57	16.73 ± 0.21	40.53 ± 1.59	50.95 ± 1.27	18.05 ± 0.78	32.71 ± 0.23	93.90 ± 1.61	4.58 ± 0.29	8.31 ± 0.12	0.28
S10	2.74 ± 0.12	14.39 ± 0.15	23.33 ± 0.77	6.13 ± 0.35	7.11 ± 0.21	13.74 ± 0.15	84.91 ± 3.74	1.35 ± 0.08	2.98 ± 0.06	0.13***^, ###^
S11	10.16 ± 0.18	15.77 ± 0.33	22.35 ± 0.96	3.17 ± 0.12	4.36 ± 0.13	13.86 ± 0.13	77.04 ± 1.23	1.71 ± 0.04	0.39 ± 0.03	0.09***^, ###^
S12	11.39 ± 0.35	9.34 ± 0.09	6.25 ± 1.23	69.94 ± 3.70	8.28 ± 0.17	38.08 ± 0.59	92.59 ± 2.55	26.50 ± 1.70	63.87 ± 0.73	0.3
S13	–	11.57 ± 0.67	7.30 ± 0.37	46.27 ± 0.98	10.18 ± 0.27	7.88 ± 0.76	83.23 ± 2.37	8.25 ± 0.63	11.74 ± 1.91	0.13***^, ###^
S14	8.48 ± 0.05	13.51 ± 0.92	37.67 ± 0.95	41.70 ± 1.32	8.18 ± 0.35	16.23 ± 0.84	90.63 ± 5.10	22.13 ± 0.51	5.99 ± 0.57	0.21^###^

– Not detected; compared with S1 group, * *P* < 0.05, ** *P* < 0.01,*** *P* < 0.01; compared with S4 group, ^#^ *P* < 0.05, ^##^ *P* < 0.01, ^###^ *P* < 0.001

**Table 3:** The O_2_^•−^ scavenging rate of the constituents from fourteen batches of the processed HSW samples (n = 6)

No.	Peak 1	Peak 17	Peak 3	Peak 5	Peak 8	Peak 18	Peak 9	Peak 11	Peak 12	Total activity (µg)
S1	30.48 ± 0.67	28.38 ± 0.15	3.69 ± 0.38	3.77 ± 0.17	5.40 ± 0.06	5.91 ± 0.28	93.88 ± 3.69	5.40 ± 0.13	10.54 ± 0.17	3.17
S2	38.70 ± 1.37	24.42 ± 0.23	–	28.43 ± 0.38	11.82 ± 0.41	13.60 ± 0.72	93.26 ± 4.52	20.84 ± 0.48	43.97 ± 0.72	3.28
S3	23.54 ± 1.57	10.79 ± 0.12	–	27.24 ± 0.23	6.50 ± 0.36	17.81 ± 0.95	87.54 ± 3.21	9.56 ± 0.58	31.16 ± 0.85	1.74^###^
S4	39.22 ± 2.36	27.74 ± 0.25	–	37.33 ± 0.95	9.76 ± 0.48	13.66 ± 0.77	96.17 ± 1.48	24.59 ± 0.88	5.94 ± 0.18	4.47
S5	40.91 ± 3.51	24.66 ± 0.49	–	3.06 ± 0.17	13.31 ± 1.07	10.96 ± 0.49	90.88 ± 3.29	4.59 ± 0.34	3.21 ± 0.24	2.32^#^
S6	23.96 ± 0.74	14.47 ± 0.65	–	15.73 ± 0.67	14.69 ± 0.52	10.30 ± 0.93	89.06 ± 1.78	7.90 ± 0.16	16.63 ± 0.36	1.87^###^
S7	30.29 ± 0.26	15.85 ± 0.37	–	13.20 ± 0.42	7.42 ± 0.21	3.96 ± 0.65	87.65 ± 2.35	3.02 ± 0.26	2.46 ± 0.72	1.62*^, ###^
S8	36.49 ± 0.48	28.09 ± 0.44	4.89 ± 0.40	5.06 ± 0.59	14.57 ± 0.67	7.75 ± 0.49	88.17 ± 2.19	3.31 ± 0.16	1.21 ± 0.53	1.8^###^
S9	51.40 ± 1.77	21.26 ± 0.68	5.71 ± 0.31	28.79 ± 0.57	8.11 ± 0.09	13.80 ± 0.13	90.57 ± 3.21	5.01 ± 0.37	5.88 ± 0.39	2.39^#^
S10	14.34 ± 0.84	15.15 ± 0.22	–	1.97 ± 0.19	2.27 ± 0.56	3.86 ± 0.76	71.00 ± 1.39	–	4.89 ± 0.65	0.51***^, ###^
S11	28.02 ± 0.73	22.06 ± 0.78	–	1.30 ± 0.17	–	4.432 ± 0.31	70.58 ± 1.21	1.96 ± 0.26	–	0.58***^, ###^
S12	28.99 ± 0.45	11.29 ± 0.67	–	45.61 ± 0.93	1.92 ± 0.29	15.73 ± 0.55	94.36 ± 1.72	13.82 ± 0.95	46.18 ± 0.91	3.67
S13	17.43 ± 0.55	19.43 ± 0.35	–	27.96 ± 1.27	3.28 ± 0.37	4.00 ± 0.56	83.99 ± 1.80	4.21 ± 0.56	9.28 ± 0.16	1.21**^, ###^
S14	39.18 ± 0.94	18.63 ± 0.42	–	24.04 ± 1.82	–	6.71 ± 0.21	87.96 ± 2.70	5.99 ± 0.27	4.71 ± 0.22	1.76^###^

– Not detected; compared with S1 group, * *P* < 0.05,** *P* < 0.01,*** *P* < 0.01; compared with S4 group, ^#^ *P* < 0.05, ^##^ *P* < 0.01, ^###^ *P* < 0.001

**Table 4:** ONOO^−^ scavenging rate of constituents from 14 batches of processed HSW samples (n = 6)

NO.	Peak17	Peak 3	Peak 5	Peak 6	Peak 8	Peak 9	Peak 11	Peak 12	Peak 13	Peak 16	Total activity (µg)
S1	49.93 ± 0.64	69.52 ± 1.24	35.73 ± 1.80	5.01 ± 0.34	43.21 ± 1.33	94.37 ± 3.45	10.40 ± 0.19	3.97 ± 0.17	6.91 ± 0.44	49.05 ± 0.93	0.14***^, ###^
S2	51.72 ± 1.22	38.09 ± 0.45	90.57 ± 3.76	25.24 ± 0.56	68.83 ± 1.31	91.49 ± 1.37	28.39 ± 0.64	19.48 ± 0.04	21.67 ± 0.88	16.35 ± 0.35	0.15***^, ##^
S3	29.07 ± 0.65	65.71 ± 0.62	92.42 ± 0.79	60.95 ± 1.33	64.41 ± 2.19	95.39 ± 1.46	12.95 ± 0.45	3.57 ± 0.20	17.26 ± 0.12	6.75 ± 0.20	0.12***^, ###^
S4	59.74 ± 2.56	67.28 ± 2.61	93.61 ± 2.99	44.33 ± 0.51	67.33 ± 0.64	95.67 ± 4.10	28.38 ± 0.46	3.85 ± 0.10	8.83 ± 0.16	47.59 ± 0.13	0.15***^, ###^
S5	46.56 ± 3.78	50.42 ± 1.33	45.81 ± 0.79	8.83 ± 0.24	72.21 ± 3.10	97.15 ± 2.08	13.15 ± 0.88	3.84 ± 0.04	7.55 ± 0.30	19.82 ± 0.25	0.13***^, ###^
S6	42.32 ± 2.19	41.31 ± 1.28	80.45 ± 1.35	20.22 ± 0.11	77.98 ± 1.12	90.65 ± 3.48	15.57 ± 0.33	–	6.80 ± 0.06	18.21 ± 0.08	0.12***^, ###^
S7	18.97 ± 0.52	26.39 ± 0.77	55.57 ± 0.23	20.50 ± 0.38	39.47 ± 0.93	87.05 ± 2.13		–	–	27.03 ± 0.42	0.12***^, ###^
S8	22.70 ± 0.72	36.84 ± 0.09	16.73 ± 0.19	5.74 ± 0.10	42.08 ± 0.67	80.86 ± 2.79	3.37 ± 0.11	–	–	21.87 ± 0.61	0.1***^, ####^
S9	12.97 ± 0.79	32.39 ± 1.88	43.80 ± 1.04	22.23 ± 0.27	20.58 ± 0.88	86.27 ± 1.41	–	–	–	14.92 ± 0.31	0.1***^, ###^
S10	–	15.44 ± 0.66	–	–	7.04 ± 0.22	64.77 ± 0.39	–	–	–	21.13 ± 0.81	0.04***^, ###^
S11	8.09 ± 0.19	34.02 ± 0.21	–	–	4.94 ± 0.79	70.21 ± 1.03	–	–	–	27.52 ± 0.13	0.06***^, ###^
S12	15.23 ± 0.35	16.94 ± 0.16	84.77 ± 0.62	19.66 ± 0.33	19.46 ± 0.44	90.12 ± 1.08	20.67 ± 0.61	14.35 ± 0.37	18.39 ± 0.07	8.11 ± 0.76	0.10***^, ###^
S13	37.66 ± 1.67	36.12 ± 0.62	88.86 ± 1.34	32.31 ± 1.75	33.76 ± 0.36	93.50 ± 0.49	13.14 ± 0.66	–	–	13.55 ± 0.46	0.12***^###^
S14	33.40 ± 1.27	80.75 ± 2.36	83.45 ± 2.35	46.39 ± 0.26	39.91 ± 0.66	92.08 ± 0.48	8.21 ± 0.90	–	12.14 ± 0.10	28.29 ± 0.95	0.13**^,###^

–Not detected; compared with S1 group, * P < 0.05,** *P* < 0.01,*** *P* < 0.01; compared with S4 group, ^#^ *P* < 0.05, ^##^ *P* < 0.01, ^###^ *P* < 0.001

**Fig. 3:**
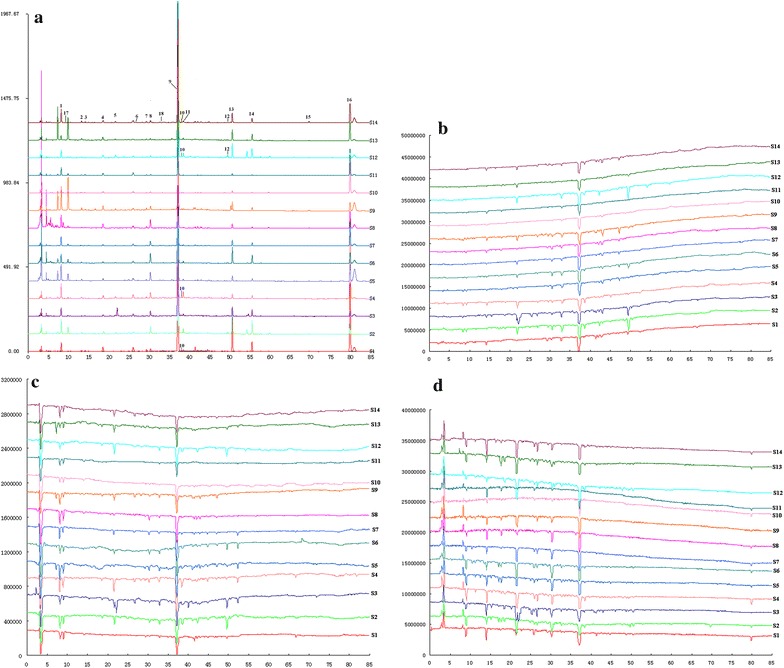
The HPLC fingerprints (**a**) of processed HSW samples from different habitats, H_2_O_2_ scavenging profiles (**b**) of processed HSW samples, O_2_^•−^scavenging profiles (**c**) of processed HSW samples and ONOO^−^scavenging profiles (**d**) of processed HSW samples (refer to Table 1 for peak numbering)

**Fig. 4:**
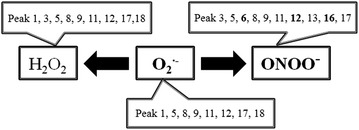
The antioxidants in processed HSW samples and the corresponding ROS antioxidative effect

In the reactive species scavenging test, stilbene glucosides, including *cis*-2,3,5,4′-tetrahydroxy-stilbene-2-O-β-d-glucoside (peak 8), *trans*-2,3,5,4′-tetrahydroxy-stilbene-2-O-β-d-glucoside (peak 9), and 2,3,5,4′-tetrahydroxy-stilbene-2-O-β-d-(2′′-galloyl)-glucoside (peak 11), were sensitive in scavenging three reactive species, and *trans*-2,3,5,4′-tetrahydroxy-stilbene-2-O-β-d-glucoside exhibited significant antioxidant activity because of its high content in processed HSW. Gallic acid (peak 1), protocatechuic acid (peak 3), catechin (peak 5), and torachrysone-8-O-glucoside (peak 12) exhibited scavenging activity on H_2_O_2_, O_2_^•−^, or ONOO^−^ (Fig. 3a–d). Compared with other components, epicatechin (peak 6), emodin-8-O-β-d-glucoside (peak 13), and emodin (peak 16) demonstrated higher selectivity in scavenging ONOO^−^ than on other two reactive species, especially emodin. These constituents had different scavenging capacities on different ROS and could affect different aspects of the aging process, such as directly scavenging free radicals, decreasing the oxidation of nucleic acid and nitration of protein tyrosine residues, and inhibiting apoptosis. In addition, peak 4 had no antioxidant activity, and peaks 17 and 18 showed certain antioxidant activity in the ROS scavenging test despite their weak ultraviolet absorption or unavailable MS messages. The relationship between constituents of processed HSW and the corresponding free radicals are summarized in Fig. 4.

Gallic acid, protocatechuic acid, epicatechin, catechin, *trans(cis)*-2,3,5,4′-tetrahydroxy-stilbene-2-O-β-d-glucoside, torachrysone-8-O-glucoside, emodin-8-O-β-d-glucoside, and emodinall exhibit pharmacological effects on the age-related pathological process [31–35]. In particular, *trans(cis)*-2,3,5,4′-tetrahydroxy-stilbene-2-O-β-d-glucoside, torachrysone-8-O-glucoside, and emodin-8-O-β-d-glucoside all significantly promote hair growth [4]. These constituents were selected as chemical markers for the quality control of processed HSW in this study. Among the constituents, *cis*-2,3,5,4′-tetrahydroxy-stilbene-2-O-β-d-glucoside, torachrysone-8-O-glucoside, and 2,3,5,4′-tetrahydroxy-stilbene-2-O-β-d-(2′′-galloyl)-glucoside were screened and proposed as chemical markers for the quality control of processed HSW. To the best of our knowledge, this is a novel research finding.

### HPLC fingerprints and similarities of samples from different origins

#### Similarities of samples

The relationship among samples was ascertained by comparing the similarity of the samples chromatographic fingerprint series. The relationship between sets of chromatographic fingerprints was analyzed by comparing the similarity between the objects and the reference fingerprints. The similarity values of 14 batches of samples (S01–S14) were 0.989, 0.990, 0.990, 0.989, 0.982, 0.995, 0.995, 0.986, 0.970, 0.870, 0.905, 0.989, 0.945, and 0.996 (n = 6), respectively. Overall, the processed HSW samples were very similar. Compared with other samples, the similarity indexes of samples S10 and S11 were relatively lower. The different habitats and processing technology could have caused variation in the content or chemical compositions of processed HSW [14, 36].

#### Results of HCA

**Fig. 5:**
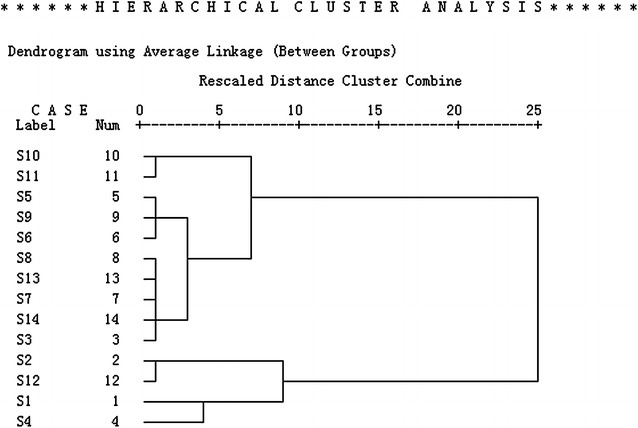
Hierarchical clustering analysis of processed HSW samples

Peak 1, peak 3, peak 5, peak 6, peak 8, peak 9, peak 11, peak 12, peak 13, and peak 16 were the antioxidants in all processed HSW samples. Peak 17 and peak 18 were not examined for weak chemical messages. HCA analysis, was performed by the SPSS statistics software. The samples of processed HSW could be classified into two clusters despite high similarity: samples S10, S11, S5, S9, S6, S8, S14, S7, S13, and S3 were in Cluster 1; samples S2, S12, S1, and S4 were in Cluster 2. The HCA results were similar to the HPLC similarity index results (Fig. 5). The data indicated that these selected constituents were the chemical markers for the quality control of processed HSW.

#### Results of PCA

**Fig. 6:**
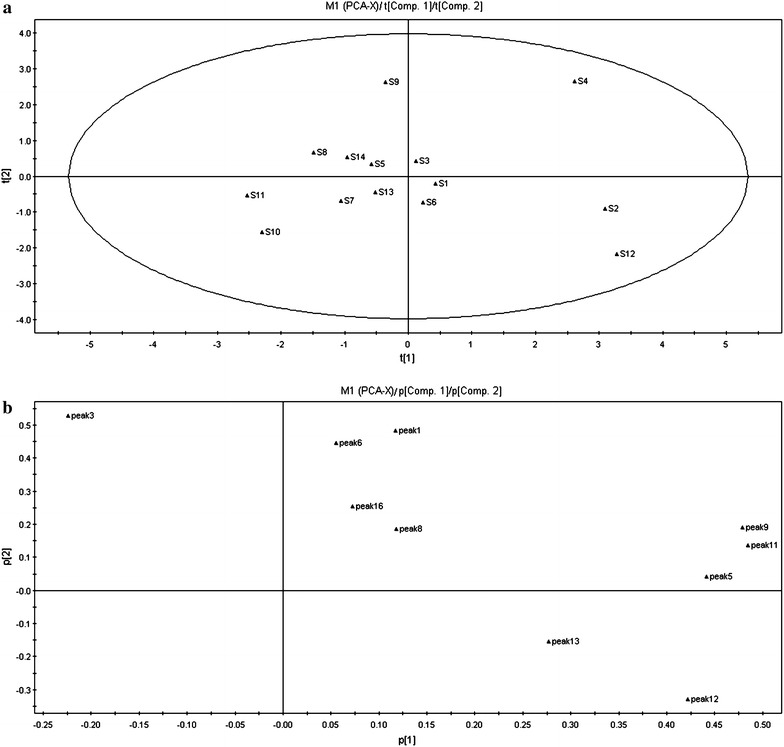
PCA Scores plot (**a**) for HPLC chromatogram data of processed HSW samples. Loadings plot (**b**) for HPLC chromatogram data of processed HSW samples

The PCA analysis produced a classification of samples similar to that produced by the HCA results (Fig. 6a). S1, S3, S5, S6, S7, S8, S9, S10, S11, S13, and S14 were classified into group 1; S2, S4, and S12 were classified into group 2. The results of the PCA loading plot indicated that protocatechuic acid, catechin, *trans*-2,3,5,4′-tetrahydroxy-stilbene-2-O-β-d-glucoside, 2,3,5, 4′-tetrahydroxy-stilbene-2-O-β-d-(2′′-galloyl)-glucoside, torachrysone-8-O-glucoside, and emodin-8-O-β-d-glucoside had relatively large influences on the difference between processed HSW samples (Fig. 6b). In addition to the common chemical markers used in the quality control of processed HSW, orachrysone-8-O-glucoside and 2,3,5,4′-tetrahydroxy-stilbene-2-O-β-d-(2′′-galloyl)-glucoside appeared to be chemical markers for the quality control of processed HSW.

## Conclusion

Our study established the antioxidative activity-integrated fingerprint for processed HSW and achieved the potential anti-aging constituents using the online HPLC–DAD–CL method with H_2_O_2_, O_2_^•−^, and ONOO^−^scavenging experiments.

## Additional files

10.1186/s13020-016-0087-8 The precision, repeatability and stability of processed HSW.
10.1186/s13020-016-0087-8 Chemical structures of the constituents in processed HSW.
10.1186/s13020-016-0087-8 Comparison of activities in scavenging three ROS between different HSW samples.

## Abbreviations

HSW: roots of *Polygonum multiflorum* Thunb.
HPLC–DAD–CL: high-performance liquid chromatography with diode array detector coupled with chemiluminescence detection
CM: Chinese medicine
UHPLC-LTQ-Orbitrap MS: UHPLC with linear ion trap-Orbitrap tandem mass spectrometry
RSDs: the relative standard deviations
HCA: hierarchical cluster analysis
