# Supplementary material for: Integrated chemometric fingerprints of antioxidant activities and HPLC–DAD–CL for assessing the quality of the processed roots of Polygonum multiflorum Thunb. (Heshouwu)
Source: Chin Med. 2016 Apr 12;11:18. doi: 10.1186/s13020-016-0087-8 (PMC4830048; doi:10.1186/s13020-016-0087-8)
Supplement: Supplementary file 1 — 10.1186/s13020-016-0087-8 The precision, repeatability and stability of processed HSW. [file 13020_2016_87_MOESM1_ESM.pdf]

Peak No.	Precision		Repeatability	Stability
	Intraday (n = 6)	Interday (n = 3)		
	RSD(%)	RSD(%)	RSD(%)	RSD(%)
1	0.51	1.27	0.94	0.43
2	1.67	2.36	1.32	0.89
3	0.69	1.51	2.11	1.27
4	0.95	1.06	0.88	1.21
5	2.17	1.62	2.31	1.38
6	2.33	3.97	4.10	2.8
7	2.29	3.60	3.50	0.97
8	0.71	2.66	2.87	0.44
9	2.05	2.10	1.49	0.87
11	1.80	2.55	2.19	1.3
12	2.30	1.42	3.06	2.87
13	0.67	1.38	0.97	1.16
14	1.45	1.76	0.83	0.62
15	1.65	2.36	1.99	1.13
16	2.09	1.10	3.50	1.20
