# Supplementary figures and images for: Integrated chemometric fingerprints of antioxidant activities and HPLC–DAD–CL for assessing the quality of the processed roots of Polygonum multiflorum Thunb. (Heshouwu)
Source: Chin Med. 2016 Apr 12;11:18. doi: 10.1186/s13020-016-0087-8 (PMC4830048; doi:10.1186/s13020-016-0087-8)

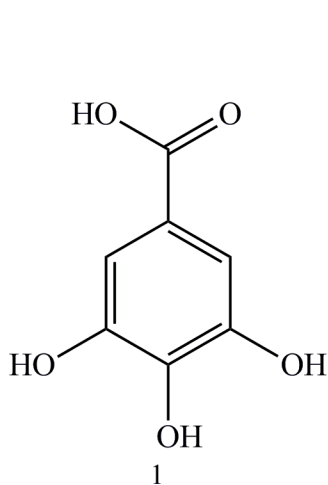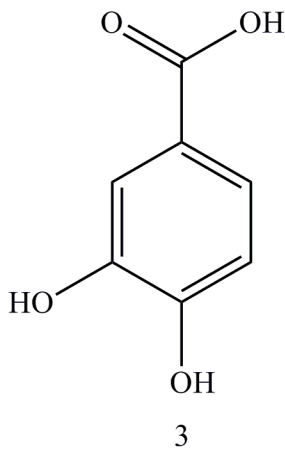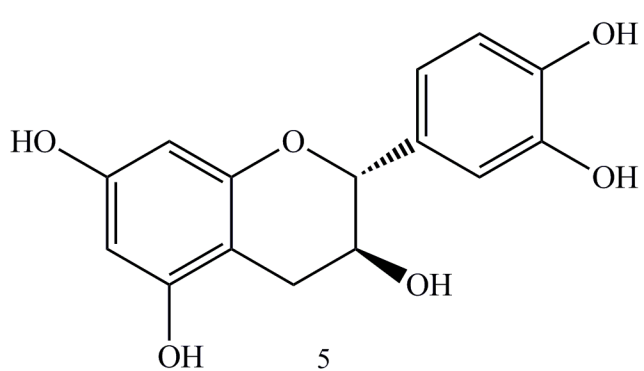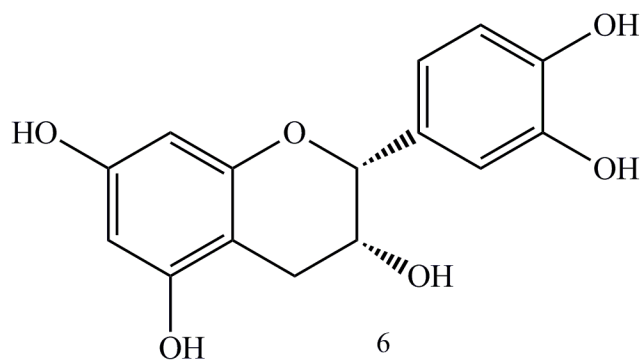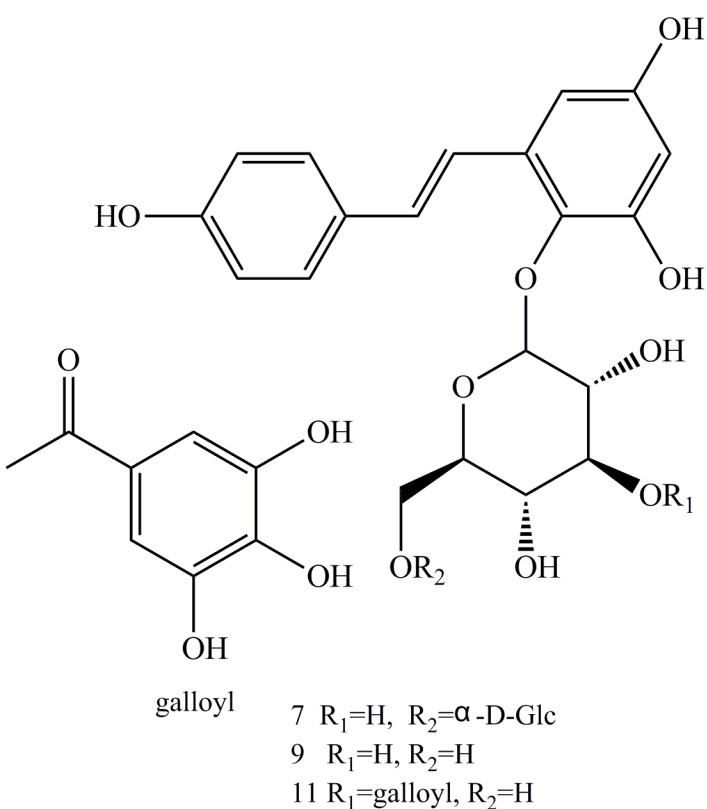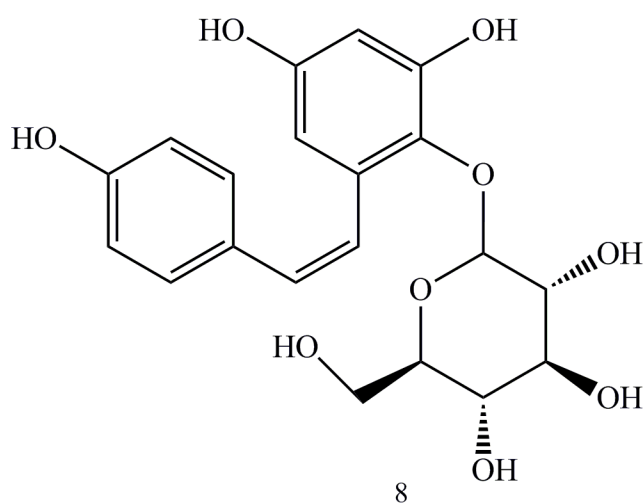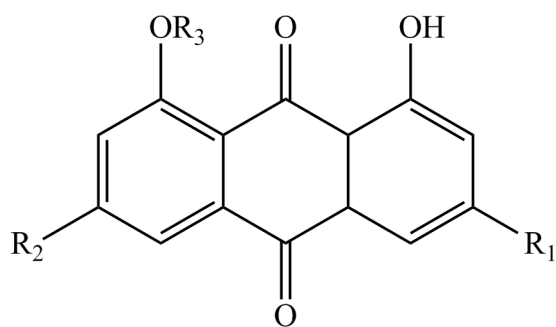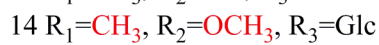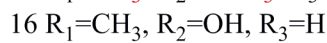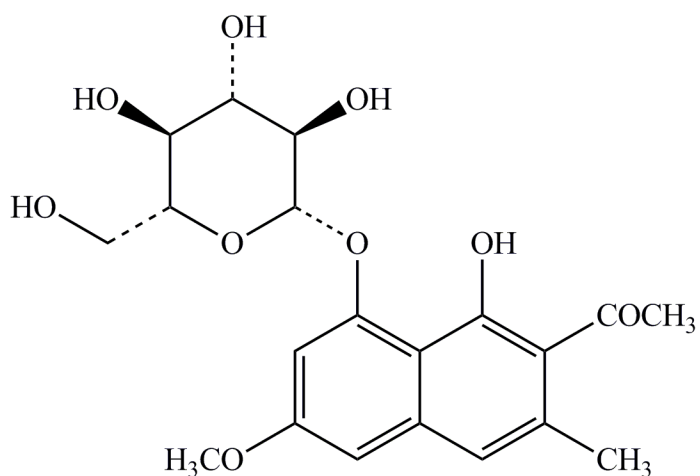

Supplement: Supplementary file 2 — 10.1186/s13020-016-0087-8 Chemical structures of the constituents in processed HSW. [file 13020_2016_87_MOESM2_ESM.pdf]
